# Anaplastic large cell lymphoma in the penis: a case report and review of literature

**DOI:** 10.1186/s13256-025-05520-8

**Published:** 2025-11-17

**Authors:** Ahmad Al-Bitar, Abd alkareem Alomar Albrazi

**Affiliations:** 1 Faculty of Medicine, Damascus University, Damascus, Syrian Arab Republic; 2 Al-Bairouni University Hospital, Faculty of Medicine, Damascus University, Damascus, Syrian Arab Republic

**Keywords:** Anaplastic large cell lymphoma (ALCL), Primary cutaneous anaplastic large cell lymphoma (pcALCL), Penis

## Abstract

**Background:**

Anaplastic large cell lymphoma is a distinct subtype of mature T cell lymphoma characterized by strong CD30 expression. While primary penile lymphomas are rare, they are most commonly of B cell origin, making primary T cell lymphomas of the penis an exceptional finding that can pose significant diagnostic challenges.

**Case presentation:**

A 60-year-old Arab male presented with multiple, rapidly growing nodules on the penile body that had developed over 4 weeks. A comprehensive workup, including biopsy and immunohistochemistry, confirmed a diagnosis of localized, anaplastic lymphoma kinase-negative primary cutaneous anaplastic large cell lymphoma. Staging via positron emission tomography–computed tomography revealed no systemic involvement. The patient was treated with six cycles of brentuximab vedotin plus cyclophosphamide, doxorubicin, vincristine, and prednisone, achieving a complete metabolic response (Deauville score of 2) as assessed by post-treatment positron emission tomography–computed tomography imaging.

**Conclusion:**

This case highlights the diagnostic challenge posed by rare penile lesions and, more importantly, the therapeutic dilemma of selecting an appropriate treatment for a disease that is localized yet multifocal. The successful outcome with systemic brentuximab vedotin plus cyclophosphamide, doxorubicin, vincristine, and prednisone therapy, a deviation from standard guidelines for unifocal primary cutaneous anaplastic large cell lymphoma, underscores the need for individualized treatment strategies and further research to establish risk-adapted guidelines for this rare clinical entity.

## Background

Anaplastic large cell lymphomas (ALCLs) comprise a category of mature T cell lymphomas that are CD30^+^ and share morphological and immunophenotypic characteristics, yet exhibit diverse clinical and genetic features [1].

Globally, ALCLs of all types constitute approximately 15% of peripheral T cell lymphomas. These lymphomas are characterized by their robust expression of CD30 (Ki-1) [2–4].

In the USA, the incidence rate of ALCL is estimated at 0.25 cases per 100,000 individuals, accounting for 3–5% of all non-Hodgkin lymphomas [5, 6].

Malignancies of the penis are uncommon, with squamous carcinoma being the most prevalent type. This cancer represents less than 1% of all male cancers and 0.1% of cancer-related deaths in the USA [7, 8].

Lymphomas originating in the male reproductive system are exceptionally rare, comprising less than 5% of extranodal lymph nodes [9].

Owing to the rarity of penile lymphomas, clinicians often fail to consider this possibility when evaluating potential etiologies of a penile mass.

In this context, we present a case involving a 60-year-old male who presented with a lesion on the penile shaft, which was subsequently diagnosed as anaplastic large cell lymphoma. To our knowledge, this represents the third reported instance of ALCL in the penis.

## Case presentation

A 60-year-old Arab male presented to our department with complaints of multiple nodules on the penile body that had developed gradually over 4 weeks without any associated urinary symptoms. The patient is a farmer, a nonsmoker, does not consume alcohol, is married, and has nine children. He has no significant medical or family history and no history of exposure to chemical or radiation substances. Upon inquiry, no systemic “B” symptoms such as fever, night sweats, or unexplained weight loss were reported.

**Fig. 1:**
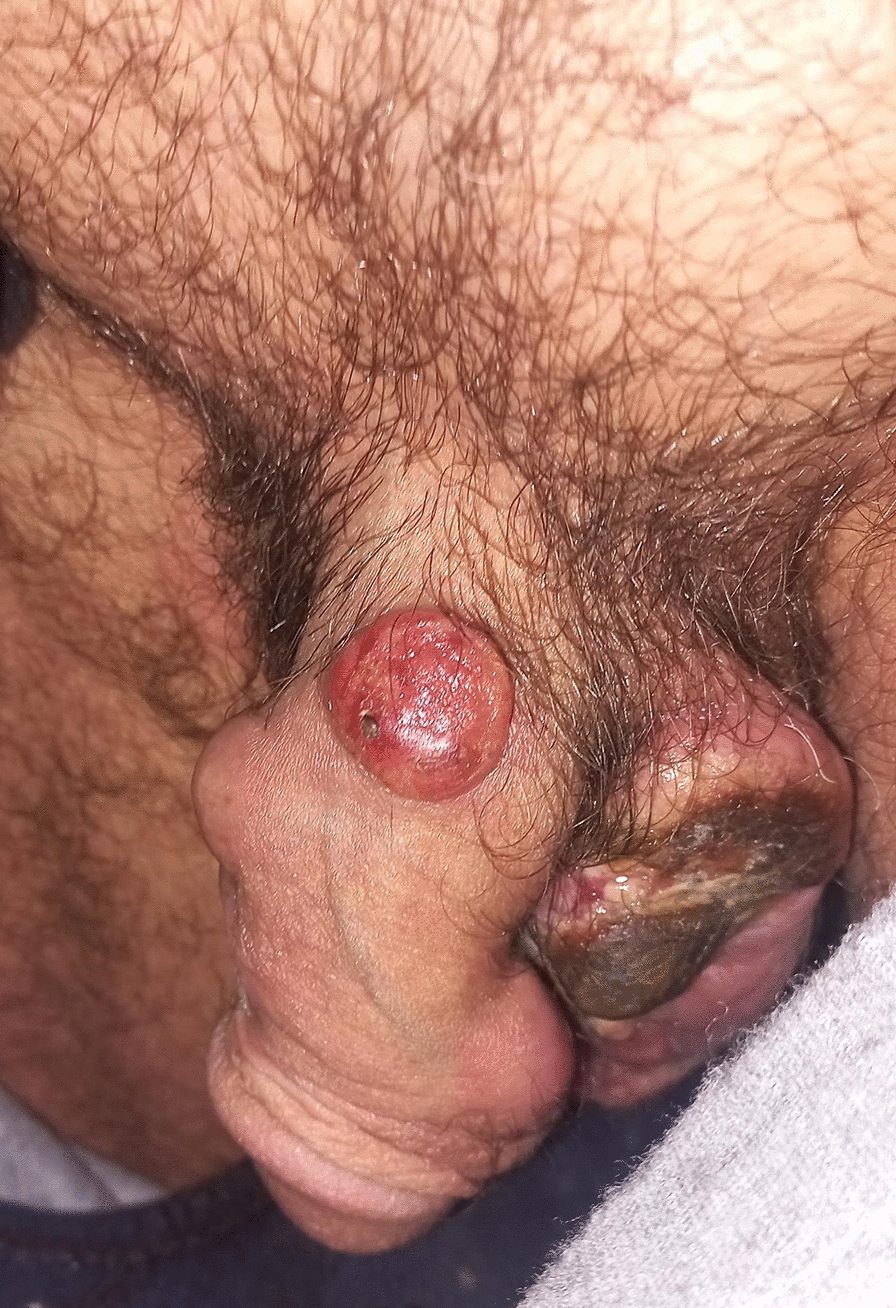
Clinical presentation at diagnosis showing multiple nodules on the penile shaft

Clinical examination revealed no palpable inguinal or pelvic lymphadenopathy, only several firm, nontender nodules on the body and base of the penis, with the largest measuring 3 cm in size (Fig. 1). A urology consultation recommended a lesion biopsy, laboratory tests, and urinalysis. The laboratory tests and urinalysis were within normal limits except for an elevated lactate dehydrogenase (LDH) of 514 U/L and an erythrocyte sedimentation rate (ESR) of 26 mm/hour.

**Fig. 2:**
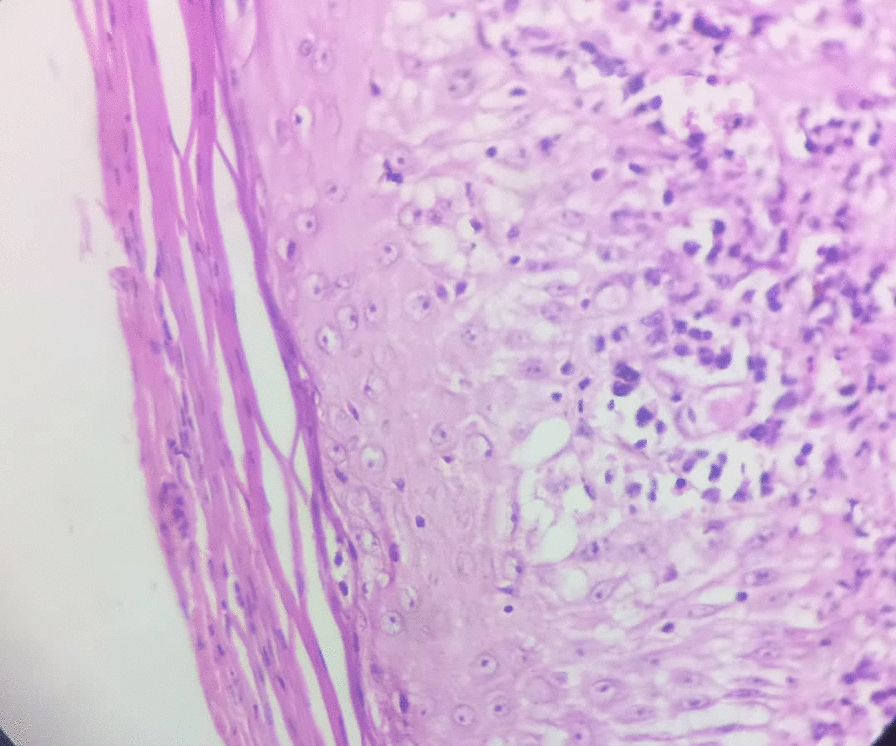
Histopathology showing a diffuse dermal infiltrate of large, anaplastic lymphoid cells (hematoxylin and eosin, 200×)

**Fig. 3:**
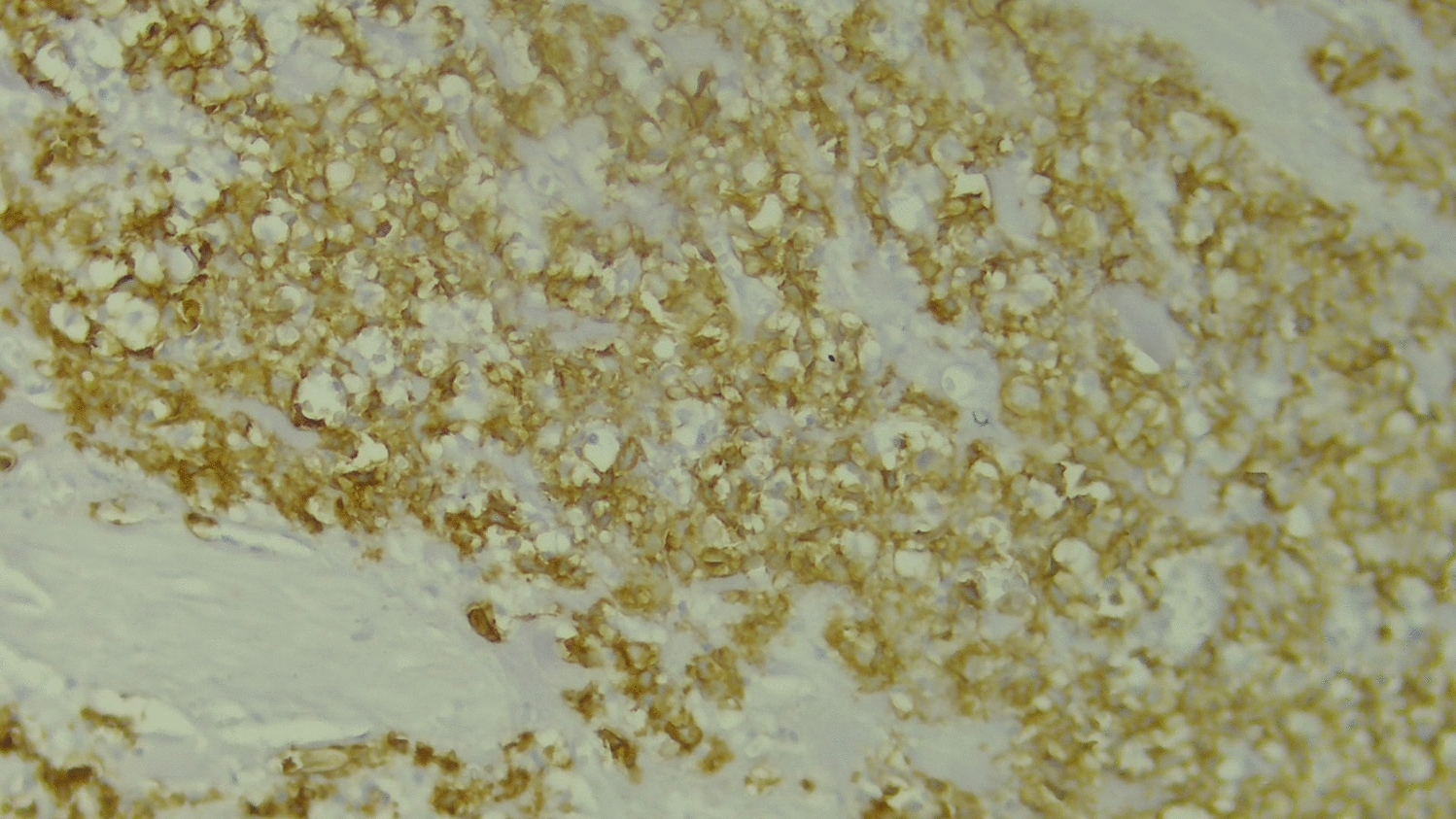
Immunohistochemistry showing strong and uniform membrane positivity for CD30 in tumor cells (400×)

**Fig. 4:**
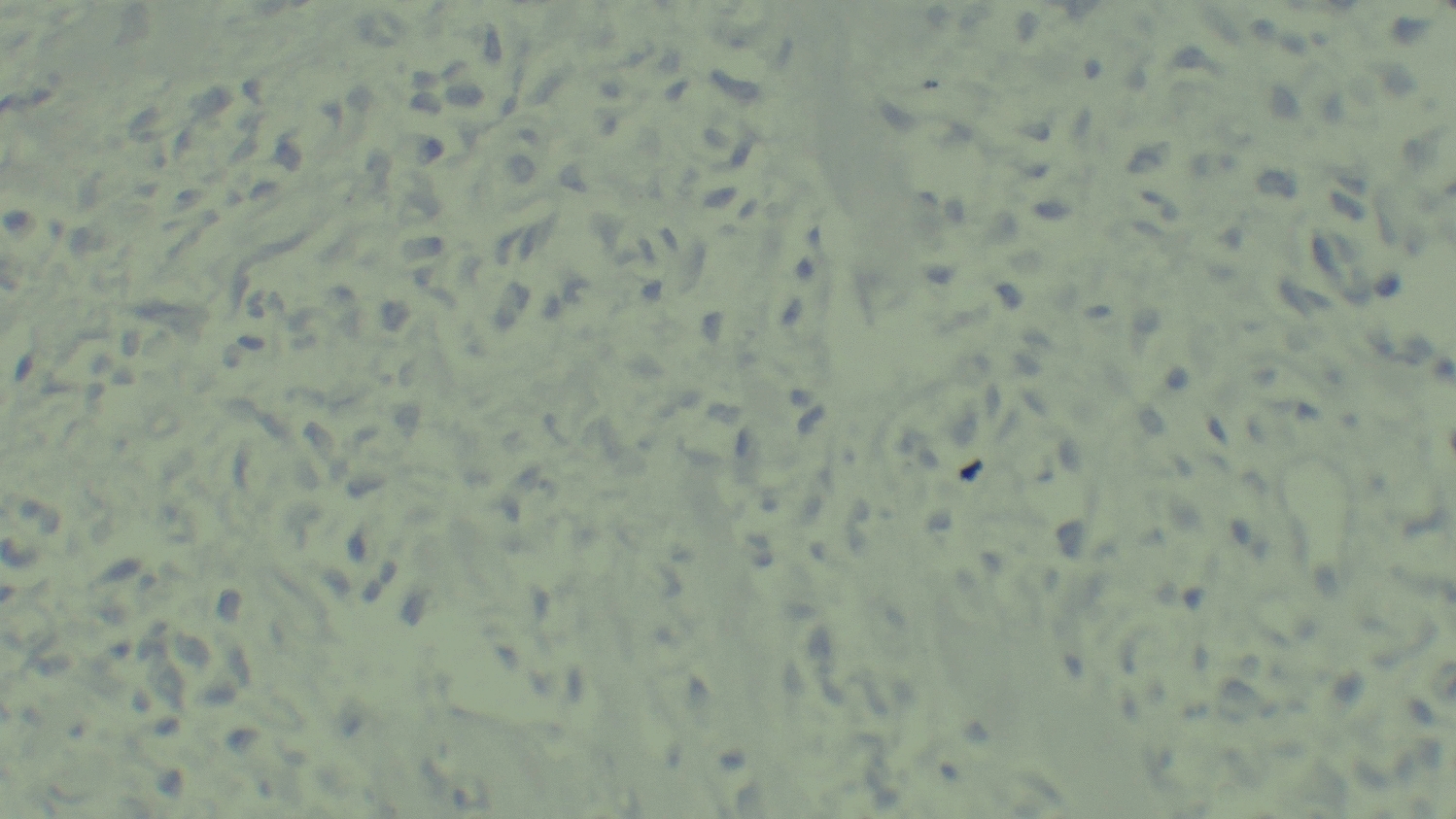
Immunohistochemistry showing tumor cells are negative for leukocyte common antigen (400×)

**Fig. 5:**
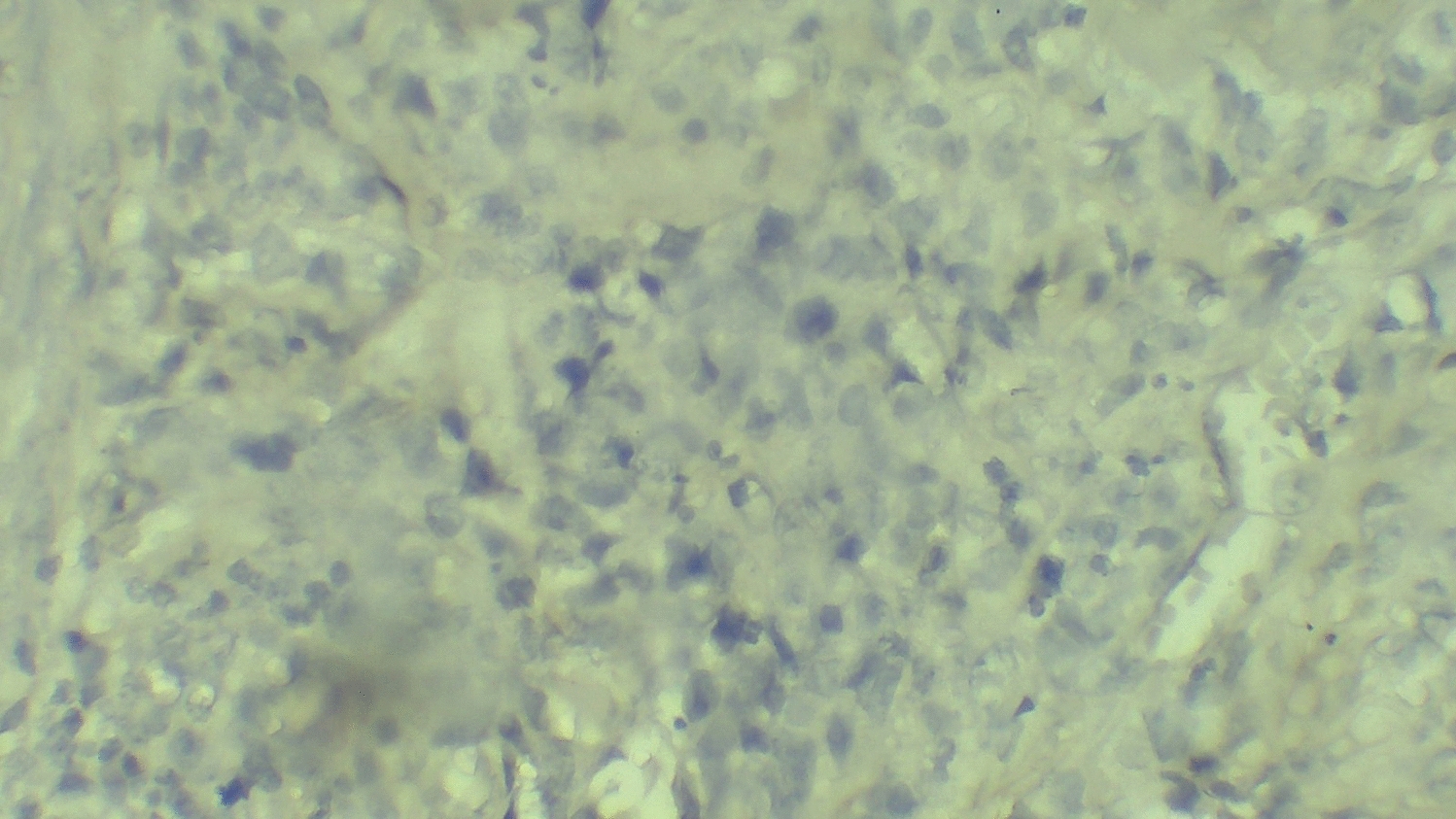
Immunohistochemistry showing tumor cells are negative for epithelial membrane antigen (400×)

**Fig. 6:**
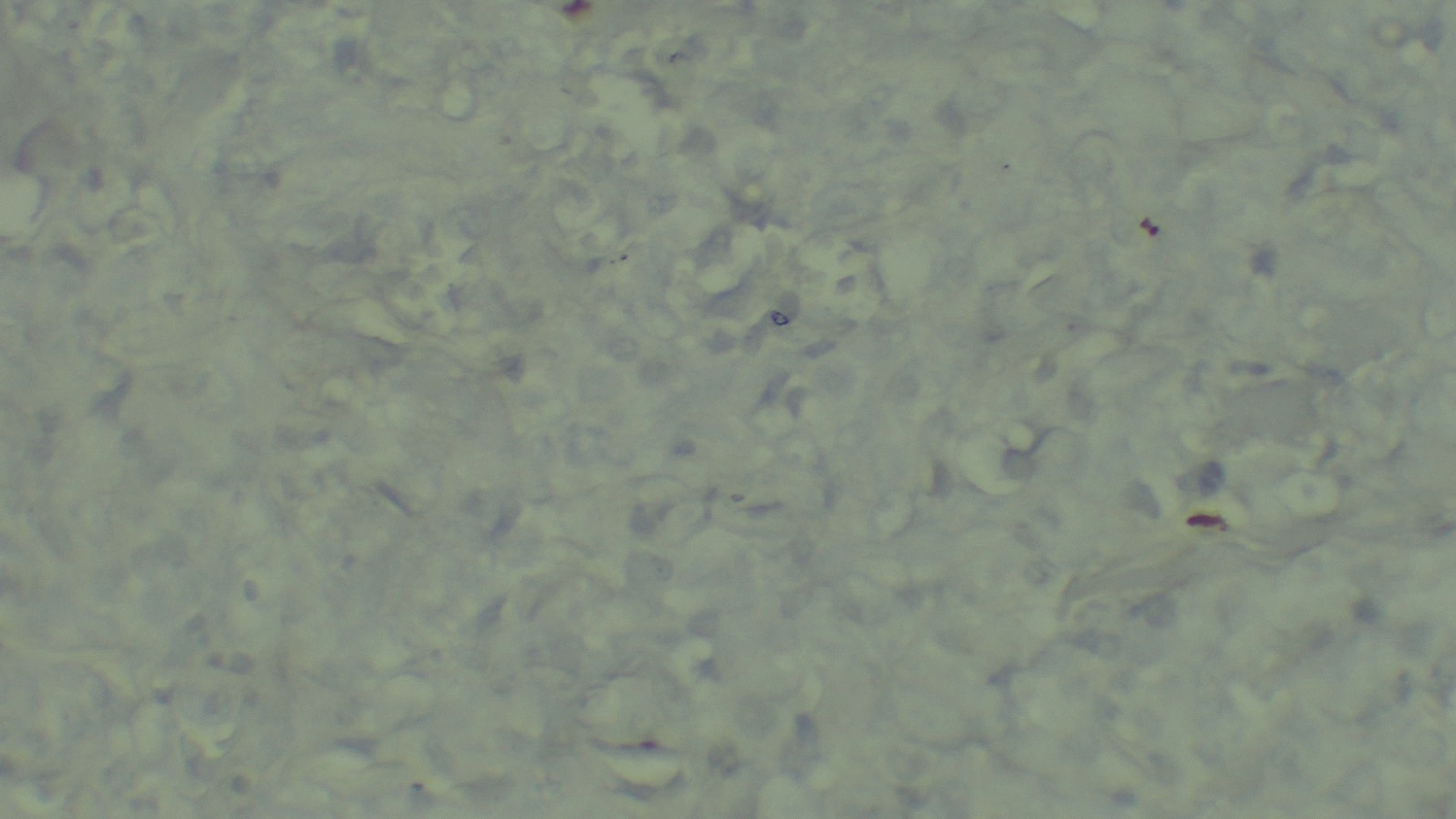
Immunohistochemistry showing tumor cells are negative for anaplastic lymphoma kinase (400×)

The histopathology report of the biopsy specimen revealed a diffuse dermal infiltrate of large, pleomorphic lymphoid cells with irregular, often kidney-shaped nuclei and prominent eosinophilic nucleoli, consistent with high-grade non-Hodgkin lymphoma (Fig. 2). Immunohistochemical staining showed that the tumor cells were widely positive for CD30 (Fig. 3) but negative for epithelial membrane antigen (EMA), leukocyte common antigen (LCA), anaplastic lymphoma kinase (ALK), CD79a, and CD20 (Figs. 4, 5, and 6). These findings confirmed a diagnosis of ALK-negative primary cutaneous anaplastic large cell lymphoma (pcALCL).

The patient underwent a staging positron emission tomography–computed tomography (PET–CT) scan to evaluate the extent of the disease. The imaging results showed fludeoxyglucose-18 (FDG)-avid lesions confined to the penis with no evidence of regional lymph node involvement or distant metastasis. A subsequent bone marrow biopsy was normal, confirming that the tumor was localized to the skin without systemic involvement.

**Fig. 7:**
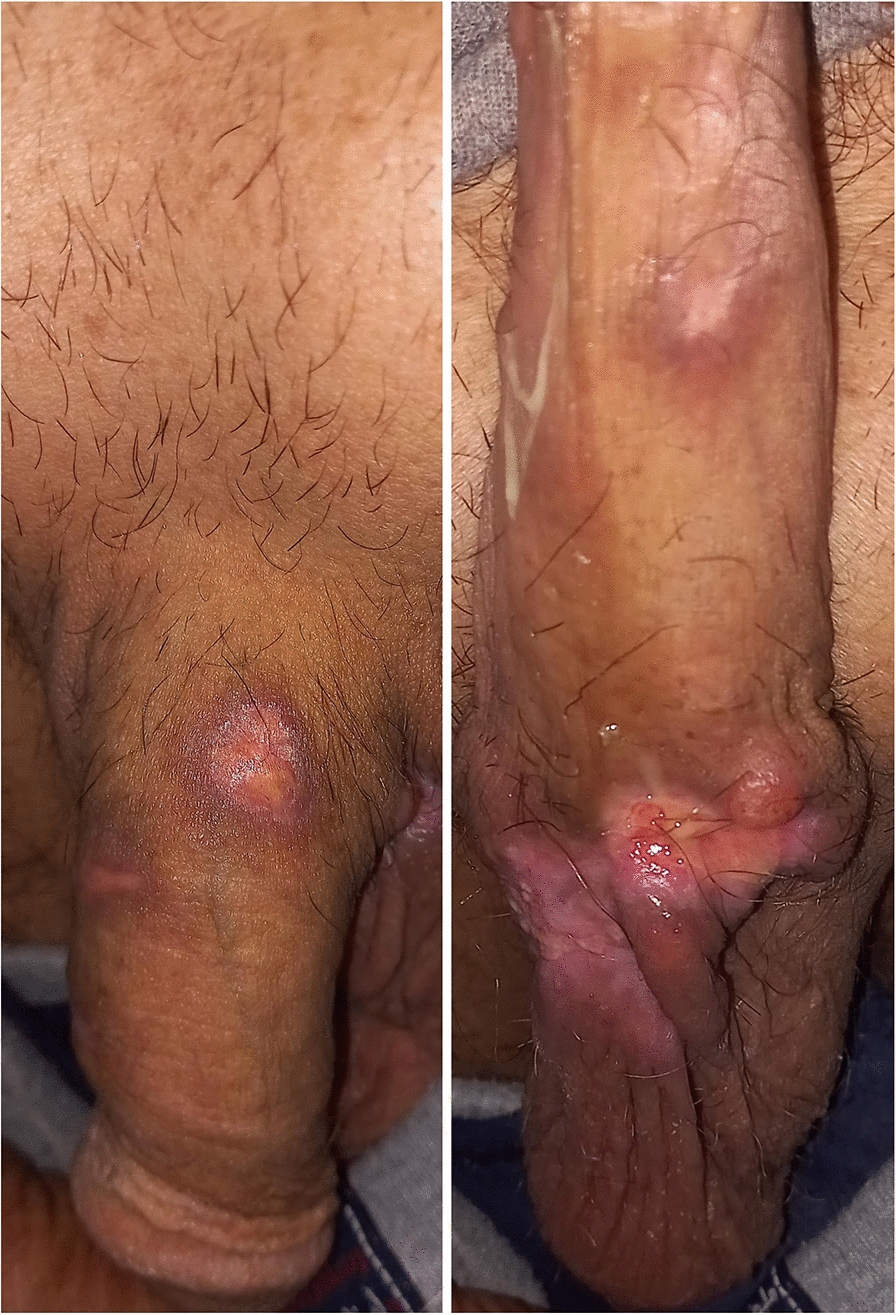
Clinical appearance after the first cycle of brentuximab vedotin, cyclophosphamide, doxorubicin, vincristine, and prednisone, showing significant regression of the nodules

**Fig. 8:**
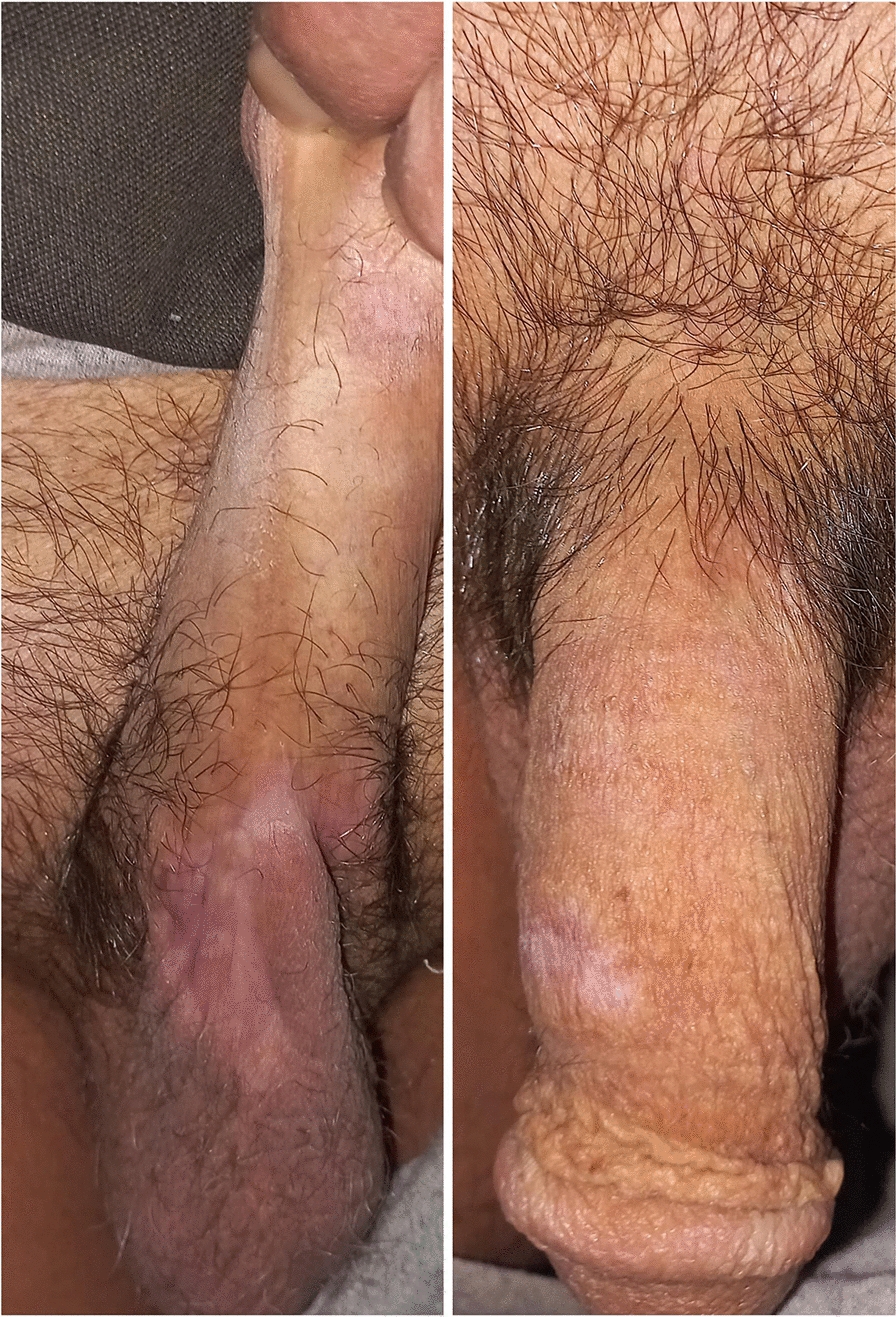
Post-treatment clinical appearance, showing significant regression of the nodules

A multidisciplinary tumor board discussed a comprehensive treatment plan. The recommended therapy included brentuximab vedotin with chemotherapy. The patient was treated with a brentuximab vedotin, cyclophosphamide, doxorubicin, vincristine, and prednisone (BV-CHOP) regimen, administered every 21 days for six cycles. The patient exhibited a significant clinical response (> 90% reduction in tumor size) after the first cycle (Fig. 7). The team closely monitored the patient for potential side effects, and the treatment was well tolerated. After completing six cycles of therapy, the patient achieved a complete metabolic response (CMR) as defined by the Lugano classification, with a Deauville score of 2 on the end-of-treatment PET–CT scan, indicating no evidence of metabolically active disease (Fig. 8). The patient remains in remission at 12 months of follow-up.

## Discussion

Anaplastic large cell lymphoma (ALCL), a distinct form of non-Hodgkin lymphoma, was initially documented by Stein *et al*. in 1985 [10]. The World Health Organization’s 2016 classification system categorizes ALCL into four types on the basis of ALK expression: systemic ALK-positive ALCL (ALCL, ALK^+^), systemic ALK-negative ALCL (ALCL, ALK^−^), primary cutaneous ALCL (pcALCL), and breast implant-associated ALCL (BI-ALCL) [11–13]. Our case represents a rare manifestation of ALK-negative pcALCL.

### Differential diagnosis of penile nodules

A key function of a case report is to educate clinicians on the diagnostic process for rare conditions, as the differential diagnosis for a penile mass is broad and requires careful evaluation to distinguish between malignant, benign, and infectious etiologies. Malignant possibilities include squamous cell carcinoma, the most common penile malignancy, which often presents as an ulcerative or fungating lesion [14–16], as well as other rare malignancies such as melanoma, Kaposi sarcoma, and metastatic disease from other primary sites [17–19]. Benign and inflammatory conditions must also be considered, such as Peyronie’s disease, which can present as a palpable, fibrous plaque, though it is typically associated with penile curvature [18] and inflammatory dermatoses such as lichen planus (violaceous plaques) and psoriasis (erythematous plaques) [19]. Finally, sexually transmitted infections are a critical differential; a primary syphilitic chancre typically presents as a painless ulcer, while condyloma acuminata (genital warts) appear as verrucous papules [19]. Definitive diagnosis in all cases of persistent penile lesions requires a biopsy with comprehensive histopathologic and immunohistochemical analysis.

### Contextualization within literature: a comparative analysis

Primary penile lymphoma is exceptionally rare, and ALCL of the penis is rarer still. A review of literature reveals only a handful of cases, highlighting the importance of each report. To contextualize our findings, we present a comparative analysis of published cases.

**Table:** 

Reference (author, year)	Patient age (years)	Clinical presentation	ALK status	Disease extent	Treatment administered	Outcome
**Present case**	60	Multiple penile nodules (largest 3 cm)	ALK-negative	Localized (pcALCL)	Brentuximab vedotin + CHOP (× 6 cycles)	Complete metabolic response; alive at follow-up
Luo *et al*., [14]	54	Recurrent penile ulcer; gastrointestinal symptoms	ALK-negative	Systemic (penis, colon, lymph nodes)	CHOP (× 1 cycle)	Died within 1 month (septic shock)
Li Chu *et al*. [15]	72	Two masses on the glans penis, initially presenting without B symptoms or lymphadenopathy	Not specified	Stage IE diffuse large B cell lymphoma of the glans penis	Resection; CHOP chemotherapy; interferon a-2b immunotherapy; rituximab (R)-CHOP chemotherapy; prednisolone and rituximab	Died due to treatment ineffectiveness on 22 February 2009. Developed brain metastases. Overall survival was 25 months
Fairfax *et al*. [20]	18	Painless penile ulcer for 7 months, gradually enlarged to 3 cm × 5 cm, occupying the entire dorsum and half the circumference. Also had daily fever (B symptoms)	Not reported	Stage IE (single extra lymphatic site) diffuse large cell immunoblastic lymphoma extending into the corpora cavernosa	8 courses of cyclophosphamide, doxorubicin, vincristine, and prednisone (CHOP)	Free of recurrence 27 months after diagnosis. Ulcer healed with scarring but with normal erectile function

This summary illustrates the heterogeneity in presentation, disease extent, and outcomes, reinforcing the lack of a standardized approach.

### Therapeutic considerations and rationale for systemic chemotherapy

The choice of BV-CHOP for what was staged as localized pcALCL represents a significant deviation from standard practice and warrants a detailed justification. For solitary or localized lesions of pcALCL, the consensus first-line therapies are local modalities, such as surgical excision or radiation therapy [21–28]. These treatments are highly effective, achieving complete remission in approximately 95% of patients with localized disease [29].

Our decision to pursue aggressive systemic chemotherapy was made by a multidisciplinary team and was based on several high-risk features present in this case. The patient presented with multiple distinct nodules on the penis, with the largest lesion measuring 3 cm. This multifocal presentation, considered a higher-risk feature for which systemic therapy may be an appropriate consideration [30], combined with a large tumor burden, increased the risk of recurrence. Furthermore, the specific anatomical location and multifocality of the lesions made local control with surgery or radiation challenging. Surgical excision would have required significant penile resection, and radiation therapy would have carried a high risk of long-term morbidity, including fibrosis, urethral stricture, and erectile dysfunction. Given these factors, the decision was made to use a highly effective systemic regimen, BV-CHOP, typically reserved for systemic ALK-negative ALCL, with the goal of achieving a durable remission while preserving organ function [31].

### Incorporating advanced prognostic and molecular insights

ALK status is a critical prognostic marker in ALCL, with ALK-negative disease generally having a poorer prognosis than ALK-positive disease [1]. ALK-negative ALCL results in a worse prognosis than ALK-positive ALCL, with 5-year survival rates of 50% and 70%, respectively [32]. However, the field is moving beyond this simple dichotomy. ALK-negative ALCL is not a monolithic entity. Gene expression profiling (GEP) studies have identified distinct molecular subgroups within ALK-negative ALCL and the broader category of peripheral T cell lymphomas (PTCL) [32]. For example, some studies have identified subgroups on the basis of the expression of transcription factors such as GATA3 and TBX21, which correlate with different clinical outcomes [33]. While such advanced molecular testing was not performed in this case, acknowledging its existence and potential value adds significant scientific depth and highlights a direction for future investigation.

## Conclusion

This case highlights the critical importance of including lymphoma in the differential diagnosis of penile masses and underscores the therapeutic dilemma faced when managing a disease that is localized yet multifocal. Our decision to employ systemic therapy with BV-CHOP, a deviation from standard guidelines for unifocal pcALCL, was driven by the multifocal presentation and was successful in achieving a complete metabolic response. The limitations of this report are its single-patient nature, the lack of long-term follow-up to assess for late relapse, and the absence of advanced molecular profiling. This case contributes to the sparse literature on this rare entity. Further research, ideally through multicenter registries, is imperative to develop standardized, risk-adapted treatment guidelines for primary penile ALCL.

## Data Availability

Data sharing does not apply to this article as no datasets were generated or analyzed during the current study.
